# A Methodology Based on Expert Systems for the Early Detection and Prevention of Hypoxemic Clinical Cases

**DOI:** 10.3390/ijerph17228644

**Published:** 2020-11-20

**Authors:** Alberto Comesaña-Campos, Manuel Casal-Guisande, Jorge Cerqueiro-Pequeño, José-Benito Bouza-Rodríguez

**Affiliations:** Department of Design in Engineering, University of Vigo, 36208 Vigo, Galicia, Spain; jcerquei@uvigo.es (J.C.-P.); jbouza@uvigo.es (J.-B.B.-R.)

**Keywords:** respiratory diseases, coronavirus disease 2019 (COVID-19), hypoxemia, medical algorithm, expert systems, decision support systems, design science research

## Abstract

Respiratory diseases are currently considered to be amongst the most frequent causes of death and disability worldwide, and even more so during the year 2020 because of the COVID-19 global pandemic. Aiming to reduce the impact of these diseases, in this work a methodology is developed that allows the early detection and prevention of potential hypoxemic clinical cases in patients vulnerable to respiratory diseases. Starting from the methodology proposed by the authors in a previous work and grounded in the definition of a set of expert systems, the methodology can generate alerts about the patient’s hypoxemic status by means of the interpretation and combination of data coming both from physical measurements and from the considerations of health professionals. A concurrent set of Mamdani-type fuzzy-logic inference systems allows the collecting and processing of information, thus determining a final alert associated with the measurement of the global hypoxemic risk. This new methodology has been tested experimentally, producing positive results so far from the viewpoint of time reduction in the detection of a blood oxygen saturation deficit condition, thus implicitly improving the consequent treatment options and reducing the potential adverse effects on the patient’s health.

## 1. Introduction

It is commonly accepted nowadays that respiratory diseases are amongst the most frequent causes of death and disability and, more specifically, they are ranked in the fifth position of the list of main causes of death [1,2]. Furthermore, it is necessary to mention the exceptional situation that the world population has been exposed to during the year 2020 because of a virus outbreak, with effects associated with respiratory pathologies, commonly known as coronavirus disease 2019 (COVID-19), first registered in December 2019 in Wuhan (China) [3,4,5]. The World Health Organization (WHO) declared the global outbreak to be a “Public Health Emergency of International Concern (PHEIC)” on 30 January 2020 and later, on 11 March, a “pandemic” situation [4]. This type of situation brings together generally the establishment of home quarantine restrictions for the potentially infected patients and their close contacts in order to reduce the transmission of the virus, among other measures. In the light of this impact, a clear trend has been observed in the last years towards improving the diagnosis process for that type of diseases, aiming not only to reduce detection time but also to optimize the patient’s care and treatment, while at the same time reducing as much as possible the negative effects on their health that come mainly from late diagnosis and inappropriate treatments [1]. In this way, a new field has opened where the differential contribution lies in having methodologies available to support the process for the detection of hypoxemic cases. This is where new tools are needed that allow quick detection of those patients at risk so that early alerts are generated if factors commonly associated with blood oxygen saturation deficit are shown.

The new technologies make available a set of tools and mechanisms that allow monitoring, registering, and processing the information exchanged between doctors and patients accurately. All that constitutes a powerful tool, from the viewpoints of both the information handling and its storage and processing, that makes its integrated use possible within decision-support systems as applied to healthcare. Thornett (2001) [6] said that systems provide assistance at two levels: by supporting the medical team decisions, and by providing answers to recurrent medical issues. Rule-based systems are commonly used for the first level, while expert systems are usually deployed for the second level. These systems allow, by using different mathematical approaches, to process the collected information in order to infer or to determine some conclusions or recommendations to support decisions involved into a higher-level system [7,8]. In the present work, an adaptation and an evolution of the methodology proposed by the authors in Casal-Guisande et al. (2020) [9] is developed, applied to the prevention and early detection of hypoxemic clinical cases. Taking into consideration the information collected from different sources, both quantitative (such as sensors that monitor the patient’s health signs) and qualitative (as the interpretations from a medical expert can be), the methodology is capable of detecting potential hypoxemic clinical cases in patients at risk. In turn, this process is based on concurrent Mamdani-type fuzzy-logic inference systems that act together and almost simultaneously to provide an effective support to the inference and decision-making process. A subsequent analysis produces hypoxemic-risk alerts that aim, not only to improve the assessment process, but also to improve the subsequent treatment in order to avoid potential negative effects on the patient’s health associated with late detection.

This work is organized as follows. In the current section (Section 1), the basic concepts needed for the definition and understanding of the methodology are presented, from both the engineering and healthcare points of view. Additionally, a succinct revision is made of the main research lines and contributions documented in the scope of the detection of hypoxemic clinical cases. In Section 2, an introduction and description of the methodology’s conceptual design is made through the presentation of the different stages and events which it follows across. After that, the methodology is formally defined and developed. In Section 3, a practical case is presented to show the application of the methodology. Additionally, the results of this work are presented using several different statistical parameters for their assessment. Finally, in Section 4 and Section 5, the discussion of the work and the conclusions obtained are respectively presented.

### 1.1. Medical Evaluation Process

There are generally well-defined actuation protocols related to the medical evaluation process [10]. In regard to the common and well-known processes for the diagnosis and detection of clinical cases of respiratory insufficiency due to oxygen deficit, the definition of hypoxemia, the usual approach involves following the guidelines or protocols indicated in the applicable procedures [11,12]. Even though these procedures demand in-person interaction with the patient, their foundations can be translated to any semi-automated evaluation process, where the following stages should be followed [11]:

1. Search for information on the patient’s health status.
2. Assessment of the collected information.
3. Development of a treatment plan.

The aforementioned evaluation process uses as a backbone the data available related to the patient’s health status, which can be grouped according to two classes [11]:

- Subjective data: symptoms.
- Objective data: signals.

Therefore, it is the processing of the collected data what makes possible that the medical expert produces a diagnosis, and therefore a consideration about the best treatment. It must be highlighted the value of the patient’s health history as a useful source of information, as it contains a detailed chronology on her health status from birth to the present. Furthermore, all considerations related to the patient’s background—such as family medical histories, work/home environment, etc.—must also be considered into the process [11,12]. Besides that, the information associated with any potential risk of contact with people diagnosed with COVID-19 is a matter of interest, with regard to visiting high virus incidence zones. 

### 1.2. Use of Expert Systems in Decision-Support Tools

Any process where decisions are made from the interpretation of information may be contained in an information system, and because of that it may be likely to be handled by means of tools that optimize the processing, elaboration, and differentiation of such information [13].

Inside those information systems, which might be themselves of different nature (engineering, social, health, etc.), tools are defined and developed that allow to provide support to the making of decisions, an activity that is implicit in the information processing in itself. Within this framework, the decision-making process allows to combine and to hierarchize the information in a way that makes possible to extract new, more elaborate information that will provide an effective support to decision-making. In the search for—and the definition of—systems that articulate that procedure, from data collection through data processing, decision-support tools appear that are backed by expert systems. These systems, initially developed in the 1960s [8,14,15], are computer applications that can transfer human knowledge and experience into software [8,16,17]. In this way, and by means of different algorithms and inference techniques, they are able to provide solutions to complex problems in a way that is similar to how an expert would do it [7,18,19,20,21,22]. By using the potential of these systems for the treatment and resolution of problems, its applicability is found within decision theory and information systems as complementary decision-support tools [23,24,25,26,27,28]. Expert systems need for their operation of several fundamental components, such as: an expert knowledge-base, the problem data, an inference system, and a human–machine interaction interface [14,15,16,17,29,30,31]. Once they have been designed, expert systems may be grounded on different techniques and ways to represent the aforementioned components, always with the aim of transforming the information, both in its nature and in its representation, until a plausible result is provided. In recent years, many methodologies have arisen supported on expert systems, among which are the fuzzy-inference systems [21,22] applied to multi-criteria problems, where neural networks [32] are used, for example.

### 1.3. Expert Systems in Health Applications

A health supervision system is in essence an information system where a process for information capture is established, to be later processed in an appropriate way so that more elaborated and useful information may be extracted from it. Finally, decisions are made based on the latter related to diagnosis, treatment, or any other aspect of the clinical procedures. Therefore, it might be concluded that a medical evaluation process, within a larger supervision system, results in essence to be similar to a generic decision-making process, as in both cases the same schematic is presented: information is collected, it is then stored and processed, and it is consequently assessed according to certain criteria that allow to establish the health status of the patient by means of the appropriate decisions. In this sense, Thornett [6] identifies expert systems as one of the two types that, together with rule-based systems, are applicable to medical decision systems, focusing their use towards those applications in which it is necessary to solve or anticipate issues in this field. On the other hand, Schröder [33] performs a revision of the decision support systems for medical diagnosis, including expert systems as complementary to decision-making. This work points to the complementary role of the clinical decision support systems, highlighting the existing debate with regard to their meaningful usefulness within the diagnosis process. As complementary tools, expert systems may—and must—carry out tasks to help and support the clinical diagnosis of diseases, as they provide capabilities and functionalities that, when properly focused, may be of assistance to the healthcare team while reducing the reticence and distrust regarding their use.

By way of example, there are nowadays different applications, either commercially available or undergoing development, that translate the use of expert systems into the medical/healthcare field [34,35,36]. Among them, those used within the United States of America’s Medicare plan, such as the LDS system [37], may be highlighted. Other applications similar to that one can be derived from scientific articles such as the proposed by M. Campos et al. [38] in which a methodology is defined to help in the selection of antibiotics therapies, or the one proposed by Yanpeng Qu et al. [39] in which a methodology is established to help in the evaluation of breast cancer risk. 

### 1.4. Current Approaches for the Detection of Hypoxemic Clinical Cases

In this sub-section, a review is made of the techniques and methods used for the detection and monitoring of potential hypoxemic clinical cases, according to two main research lines. On the one hand, the use of measurement instruments for the determination of hypoxemic clinical cases is highlighted, while on the other hand it is considered the integration of the information provided by those devices and by other sources in the definition of clinical decision support systems applied to the detection and monitoring of hypoxemic cases.

#### 1.4.1. Measurement Instruments Used in the Determination of Hypoxemic Clinical Cases

The pulse-oximeter is a device that allows to assess the oxygenation status of a patient’s blood by means of a non-invasive technique in a simple and objective way [40]. It has become at this moment the main and most accepted measurement device for the recurrent assessment of blood oxygen deficiency. Its use may reduce the need for gasometries and the healthcare costs associated with these procedures [41]. The readings obtained with that device, characterized as the percentage of oxygen saturation in blood, are considered reliable within the 70–100% range, having a confidence interval of ±4% [40] at a value of 95%.

As regards to the interpretation of the results obtained, blood oxygen saturation levels within the 97–99% range indicate a normal health status, even if a normal status could be considered as well for lower saturation levels in patients with a long smoking history [40]. On the other hand, patients showing oxygen saturation levels below 90% might require supplementary oxygen administration and to perform a gasometry in order to confirm the hypoxemic state. According to Fanconi [42], when a patient shows pulse-oximeter oxygen saturation measurements lower than 75–80%, then invasive techniques must be applied to determine the actual blood oxygen level, thus allowing the detection of potential severe hypoxemic cases that might not be otherwise detected.

Different pulse-oximeter types are available that may be placed on different body regions of the patient such as: a finger, an ear, a foot, or the forehead [12]. However, the specific body area where those measurements are carried out may affect the early detection of hypoxemic conditions. The study by Hamber et al. [43] shows significant delays in the detection of hypoxemic cases when the measurement device is placed on a foot in comparison with when it is placed on an ear or on a hand. Additionally, it is important to indicate that, even in the case that the pulse-oximenter readings indicate a correct blood oxygen level, this does not imply a correct removal of the carbon dioxide in blood, and therefore such isolated measurement cannot be considered as a characterizing signal of lung ventilation performance [44]. In order to achieve an accurate characterization of the lung ventilation activity, the use of the pulse-oximeter might be combined with that of a capnograph. Another alternative approach would involve performing a gasometry, a more invasive and exceptional procedure. 

#### 1.4.2. Clinical Decision Support Systems Applied to the Detection and Monitoring of Hypoxemic Cases

With regard to the clinical decision support systems mentioned in this section, most of them share a common process based in the capture of data on the patient’s health status by means of a pulse-oximeter, and a subsequent generation of alerts based on previously defined criteria and/or ranges.

In this sense, for example, the work by Karlen et al. [45] proposes a system aimed to mobile devices that, connected to a pulse-oximeter, allows the monitorization of patients that were subject to anesthesia. The autors highlight the low cost ot this solution, which by means of a graphical interface provides the healthcare professionals with the information needed to make decisions associated to the patient status during a medical intervention. In this same line, but aimed towards a personal use, in the work by Keerthika and Ganesan [46] the design of a PDA-like device is proposed for the monitoring of blood oxygen saturation and heart rate. Additionally, the system allows the generation of alerts when some pre-established limits are exceeded. The authors establish a first limit for oxygen saturation level of 80% to warn the patient to be careful. On the other hand, if the value is lower than 50%, then a warning is sent to the healthcare staff to request immediate attention for the patient. The work by Işik and Güler [47] develops a mobile application to be used at home that feeds the readings of a Bluetooth-connected pulse-oximeter into a web database. Besides that, such system incorporates an algorithm that sends SMS alerts to the healthcare staff when the saturation level is lower than 80% or the pulse is not in the 40–150 b.p.m. range. In the work by Merone et al. [48] a decision support system is developed aimed towards the remote monitoring of COPD (Chronic Obstructive Pulmonary Disease) patients, named the “Worrisome Event Detection System”. The system is based on the use of Petri nets, which by means of several training stages allow to adapt and to customize the predictive model to each specific patient. The work by Hassan et al. [49] proposes the design of the physical components of a machine learning model based on a fully connected neural network, aimed to the detection of respiratory failure episodes in patients affected by sleep apnea at a neonatal ICU (intensive care unit). The system feeds on both data coming from a commercial pulse-oximeter and a continuous breathing signal coming from another sensor, in this case a polyvinylidene fluoride (PVDF)-based pyroelectric transducer. Once the model has been trained, it is fed the signals from the previously mentioned sensors, aiming to predict apnea events and to make possible the generation of early alerts. In the work by Ghazal et al. [50] a study is made in which it is assessed whether a machine learning-based prediction model might determine the changes in the blood oxygen saturation level after changes were made to the operation parameters of the mechanical ventilation a patient is subject to. Even if at that moment the results were not satisfactory enough, as the accuracy obtained was low, the authors concluded that the bagged complex trees approach used resulted to be the best one. Lastly, it is also of interest to mention the work by Al-Taee et al. [51], in which a tool for mobile devices is proposed that allows to provide help in the making of decisions associated to the interpretation of gasometry results.

### 1.5. Prior Considerations

On the light of the previous study and the research lines that were pointed out, in this work the adaptation and evolution of the methodology proposed by the authors in Casal Guisande et al. [9] are applied to the medical evaluation of respiratory deficit-hypoxemic cases. To do that, it will be necessary to define the management of the process information, as well as the protocols for its collection and processing. Such methodology connects with the clinical decision support systems and allows to offer an early detection of potential hypoxemic cases. When all previously stated in Section 1.4.1 and Section 1.4.2 is taken into account, the methodology will allow the integration of the advances relative to the use of pulse-oximeters, while also incorporating the knowledge derived from the previous studies with regards to risk threshold values, measurement techniques, and interpretation factors or criteria. As it will be developed across the sections in this work, the main benefit intended to be provided is precisely the clinical decision support character. This will be reinforced by means of supervising the chain for collecting, handling, and processing data, interpreting the inferred results, and showing and clarifying the results towards their possible reinterpretation by the healthcare staff.

## 2. Materials and Methods 

### 2.1. Definition of the Methodology

#### 2.1.1. Previous Considerations

As it has been mentioned before, in the current work a methodology for the detection of hypoxemic risk cases based on the use of expert systems is modified and adapted from the one developed by the authors in their work of 2020 [9]. Because of that, a methodology is to be defined that may be integrated into the medical processes information systems scope for detecting hypoxemic cases, that will provide support to the information stored on it, and to the decision-making associated to the elaboration of the diagnosis and the selection of a treatment. As a measure that guarantees the validity and the accuracy of the presented developments as much as possible, in this work the guidelines indicated by Hevner et al. [13,52] will be observed. In their 2004 work, these authors proposed a reference framework, articulated by means of a set of seven rules, where any contribution made to the information systems field can be evaluated in a comparative way. In Table 1, a revision is made of the compliance to each one of those guidelines, in reference to the methodology presented in this article.

#### 2.1.2. Conceptual Design and Description of the Methodology

The conceptual design of the methodology will begin with the identification of the needs associated to its application [53]. As it has been explained in the previous sections, its main goal is the early detection and prevention of potential hypoxemic cases in patients at risk. Therefore, the identification of the needs that the development of the methodology must satisfy, and also because of that the software artifact that supports it, must include all those needs related to the hypoxemia diagnosis process. In this way, it must be able to capture and process the objective and subjective patient data, translate them into the expert systems, and from their result obtain a risk index value associated to a potential case of blood oxygen saturation level deficit. Additionally, all this process must be implemented into the methodology in an autonomous or semi-autonomous way, thus enabling the speeding up of the detection and repetition of the process in a recursive way. In light of the current state of the pandemic associated to COVID-19, the implementation of this methodology by means of its corresponding software application must result in a streamlined and comfortable use, for both the patients and the heath staff. Even if the methodology might gain interest in pandemic situations such as the current one in 2020 due to the COVID-19, its application is neither limited nor conditioned by any aspect directly or indirectly related to the hypoxemic risk. The goal is to assess the risk of a patient being affected by a hypoxemic condition by considering a set of variables that might have an influence on it, with COVID-19 being just one of them.

**Figure 1:**
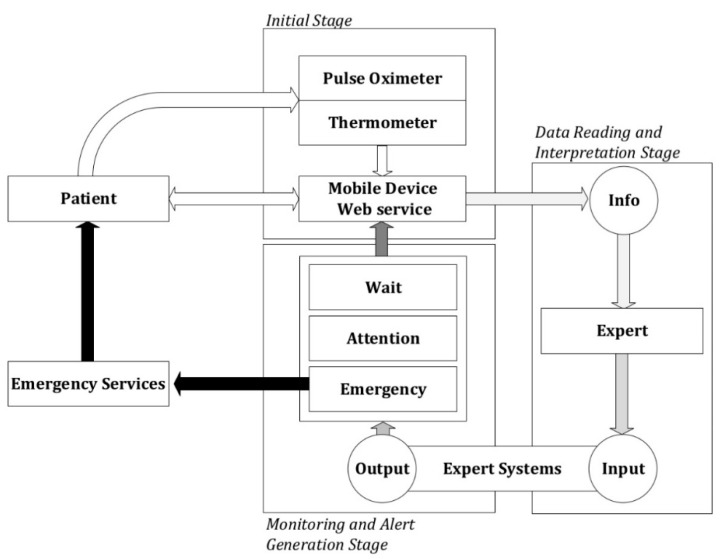
Conceptual schematic of the methodology.

Figure 1 shows a conceptual diagram of the methodology that comprehends the whole process, from detection through action. In it, the different stages for the data collection and/or processing may be observed as described in the following.

##### Initial Stage

Firstly, it is proceeded to the data collection, that will be carried out by means of a mobile application or a web service, this choice being irrelevant for the definition of the methodology. Sources for such data may be of two kinds: autonomous collection of data coming from sensor readings, and data input on the patient’s side.

Because of that, once the patient is instructed in the usage of the application, she must introduce the following data into it:

1. Personal information: name, identifier, date of birth, race, and sex.
2. Respiratory diseases: respiratory conditions the patient has been diagnosed with, such as for example chronic obstructive pulmonary disease (COPD), asthma, etc.
3. Harmful habits: If she is/is not a smoker, and in the former case for how long and how many cigarettes she usually smokes per day.

On the other hand, the patient will be asked periodically about certain risk factors that might affect her, as shown in Table 2.

This information—manually introduced by the patient—will be complemented with the other information coming from the autonomous data collection by the sensors, among which are a wireless-capable pulse oximeter and a digital thermometer. It is intended to monitor the relevant variables of the patient’s health status, such as body temperature and heart rate, together with the blood oxygen level that is directly related to respiratory conditions [11]. In this case, a question related to COVID-19 was included, because at the particular moment the methodology is being tested the COVID-19 pandemic is ongoing. This criterion is incorporated as an additional factor to be considered, but it does not condition the use of the methodology in any way.

It is important to mention that the first time the patient uses the system she will have to perform several measurement cycles with the sensors, so that the expert healthcare team may adjust the base values of the vital signs for this patient in particular.

##### Data Reading and Interpretation Stage

The second stage in the methodology is developed after the initial preparation stage, and it is carried out in parallel to it to achieve a continuous processing of data as soon as these are updated. Once the data is read and collected, they are processed by means of two concurrent expert systems [9,54] that use Mamdani-type fuzzy logic-based inference systems [55,56,57,58]. Each one of these systems performs the calculation of a risk level related to the final value of the hypoxemic risk, and they will be explained in higher detail in Section 2.2.

##### Monitoring and Alert Generation Stage

The methodology allows to perform monitoring on the patient’s health status and to generate alerts in the case it is detected that the current values lay outside the range established in the assessment process. In order to know the patient’s health status, a number of parameters must be measured with a specified frequency, in order to reduce their uncertainty. In this case, the ‘uncertainty’ concept must be understood as the variability in the accuracy—both qualitative and quantitative—of the collected data. Furthermore, besides this random conception, the management of the uncertainty derived from the lack of information is also contemplated in the methodology, due to the interaction in itself, and mainly the vagueness in the subjective assessments by the expert healthcare team [59].

Once the data has been collected and assessed within the system by means of the expert systems, an alert might be generated according to three possible levels that are associated to different value ranges for the global risk level obtained:

- Wait: It is not required to assist the patient, as it is considered that her health status does not demand it.
- Warning: It is recommended to carry out new measurements within a pre-determined period of time by the system in order to reassess the patient’s health status. The healthcare professional might provide indications about the care to be given to the patient if it is necessary.
- Emergency: It is recommended to send the emergency systems immediately to assist the patient.

It must be highlighted that these levels are always to be postulated by the expert healthcare team that is in charge of operating the proposed methodology and interpreting its results. These levels may be extended or reduced according to the expert’s criteria and implemented anew into the methodology.

### 2.2. Implementation of the Methodology

As it has been already mentioned, the authors have developed a similar methodology in a previous work [9]. That is why the implementation of the new methodology will be an adaptation of the former one to the case of hypoxemia, highlighting the similarities and differences with the model already published by the authors.

The methodology introduced above considers the collection and processing of data by means of expert systems—based, in turn, in fuzzy-logic techniques—as an efficient decision-support tool. These expert systems can process information in a way that provides an environment that makes possible to complement the decision-making process by managing, processing, and hierarchizing the collected data. Next, the behavior of said systems is to be explained. 

Starting with the data collection protocol—both from sensors and information input by the patient—the information is stored and organized to be processed by the concurrent expert systems [9,54]. In the first place, a first expert system focused on the processing of the information coming from the data collected from sensors, and in the second place a second expert system that works concurrently with the first one, and based on the criteria and opinions of an expert healthcare team derived from the assessment of the initial data.

**Figure 2:**
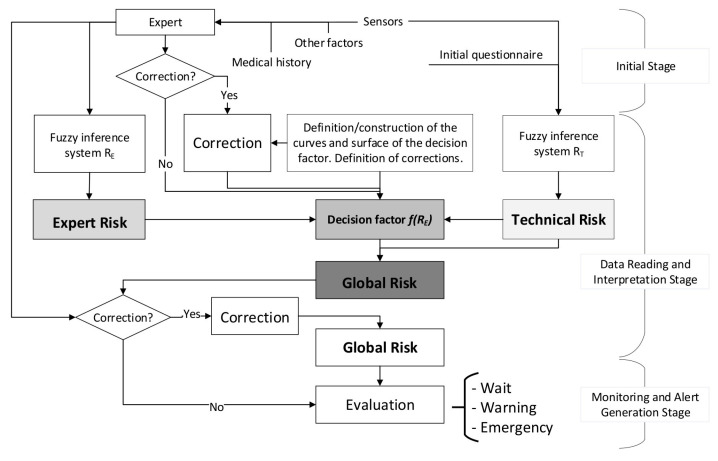
Flow chart of the methodology showing the concurrency of the two expert systems.

From the combination of the results output by both expert systems, a final alert level of the patient’s health status is obtained that corresponds to the global hypoxemic risk. Figure 2 shows the flow chart that describes the behavior of these systems, identifying the stages described in Section 2.1.2. In summary, Figure 2 shows how the expert and technical risk values are calculated first from the initial information by means of fuzzy logic-based inference concurrent systems [9,54], to be later combined to obtain a certain global risk value that allows to establish the seriousness of the patient’s health status. 

All these calculations are made by means of a piece of software built into MATLAB© R2020a [60], which allows to systematically automate the process for evaluating the patient’s health status. Broadly speaking, such automation allows to reduce the complexity of the calculation process, while at the same time facilitating its handling by the user.

#### 2.2.1. Definition of the Global Hypoxemia Risk Concept

In this work, the detection and prevention of hypoxemic cases is articulated around a set of risk index values that will result to be the output of different expert systems. These are grounded on the work developed by the authors in Casal-Guisande et al. [9], applied to the treatment of pressure ulcers, that has been appropriately modified to the case of blood oxygen saturation level issues. Because of that, the object of the methodology is to help and facilitate its detection and diagnosis. The expert systems, therefore, will determine the so-called global hypoxemic risk ($R_{G}$), i.e., a quantitative value of the patient’s risk associated to the non-intervention or non-actuation of the medical services to change—or correct—their health status, in this case focused on the low level of oxygen in blood. With that, this risk value provides a metric that allows the expert medical team to make a decision based on the combination of data, remembering that these come from both quantitative and qualitative sources. Such a decision acts on the convenience of giving the patient a treatment, hence the denomination of ‘risk’.

Equation (1) is used for the calculation of this global hypoxemic risk, making use of two previously calculated parameters: ‘technical risk’ ($R_{T}$) and ‘expert risk’ ($R_{E}$) values, these being the results provided by the two previously introduced expert systems [9].

(1) $$R_{G}\left(R_{T},R_{E}\right)\to R_{G}=\;R_{T}\cdot \frac{f\left(R_{T},R_{E}\right)}{10}\;\forall \;R_{E,}R_{T}\;\in \left[10,\;100\right]$$

The $f\left(R_{T},R_{E}\right)$ term refers to a function of the Expert Risk named as ‘decision factor’, that depends on the values of the technical risk and the expert risk, calculated from the technical and the expert assessments after their processing through the two Mamdani-type fuzzy concurrent inference systems [55,56,57,58].

A minimum value of 10 is established for the technical risk value, as in this case the global hypoxemic risk would be equal to the decision factor, which would represent the case where the technical values do not determine a decision, thus forcing the global hypoxemic risk to depend only on the expert assessment. That is the reason why the constant value ‘10’ is placed in the denominator of Equation (1).

On the other hand, the maximum value for the Technical Risk will be always of 100. For this value, the global risk will be equal to the decision factor times 10. In this case, there is a high dependence on the assessment of the objective data, resulting in an inference of a high hypoxemic risk. If the healthcare team—or the expert—wishes to reconsider the decision, she must provide significantly low expert risk valuations, which will be only accepted subject to appropriate arguments and strict justifications. The fact of considering the decision derived from the technical valuation when it results in a technical risk value of 100 will necessarily imply a great influence of the healthcare team in the decision result.

#### 2.2.2. Definition and Calculation of Technical and Expert Risk

The technical risk value is the output of the first expert system used in the definition of the methodology. This is the system in charge of assessing the technical data—from which its name is derived—that come from the sensor readings, and the type of the input data is therefore—in principle—quantitative and objective and does not require of any interpretation. Therefore, the technical risk is an inferred and quantifiable value of the potential risk on the patient’s health of the quantitative measurements of blood oxygen saturation, temperature, and personal data.

In relation to the expert risk, this is the output of the second expert system, which is in charge of the interpretation of the technical and personal data obtained during the application of the methodology, taking data of a qualitative and subjective type that requires of a higher level of interpretation. This interpretation is the responsibility of an expert healthcare team, in this case one belonging to the healthcare field. These expert evaluations are the input to the second inference system, providing as an output a specific expert risk value. Thus, same as before, this expert risk is an inferred and quantifiable value for the potential risk on the patient’s health of the collected data once it has been subject to the valuation of the healthcare expert or team as a whole.

In contrast to the first system, as it has been already mentioned, the expert assessments may be interpretative, because they derive from human language and therefore have implicitly associated a certain inaccuracy and vagueness that increases uncertainty in the process and in the final decision [9]. In this case, uncertainty will be of a mixed kind, combining the randomness of answers and metrics with the potential lack—or reduced availability—of data, and with their subjectivity. In particular, fuzzy logic-based Mamdani-type inference systems are used with the objective of keeping uncertainty under control. These systems are known to have capabilities to handle the uncertainty that is a characteristic of human language [55,56,57], and to translate the qualitative assessments into quantitative ones in order to process and represent the inaccuracy and vagueness that are inherent to it.

##### Calculation

**Figure 3:**
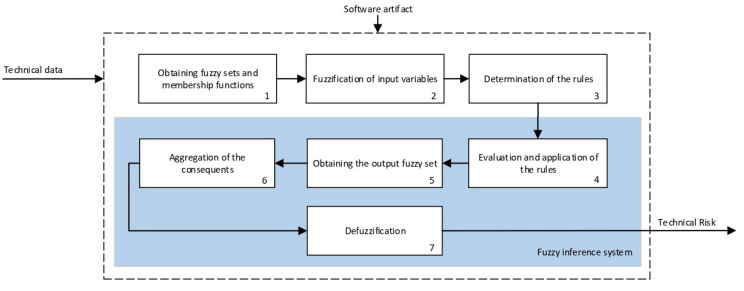
Schematic of the technical risk inference system used in the software artifact.

The technical risk and expert risk values are obtained as outputs of the defuzzification process of each inference system [58]. Figure 3 shows a schematic of the technical risk inference system, which is fully analogous to the expert risk one. Both inference systems are Mamdani-type [55,56,57,58] and they are sequentially developed, starting with an initial fuzzification of variables, combining it according to a set of pre-defined rules, in order to finally defuzzify the final output set. All that happens with the prior definition of the membership functions associated with the input and output variables.

The definition of the membership functions is performed as a previous step to the inference process. In this case, the choice for a specific type of functions is an answer to the nature and characteristics of the input variables. Starting with the general recommendation of choosing normal, convex, and symmetrical functions [58], the choice made focuses on the common triangular, trapezoidal, and Gaussian functions. As mentioned before, the choice of the form will depend on the type of input data, always looking for functions that allow to assign a degree of membership in a graphical and intuitive way [54]. There are two inference systems in this case with different input data (antecedents) and similar output data (consequents). For the technical risk system, the input data or antecedents will be obtained from sensor readings and patients’ characteristic values. For the expert risk system, the data will consist of subjective valuations founded on the interpretation of the quantitative data derived from sensors, other indicators related to the medical history of the patient, and other consideration values by the expert healthcare team. They will be in both cases objectifiable data within a certain range contained in the consideration of the expert risk because, even if the assessments are subjective, their quantification aims to be accurate. Because of this, in both systems trapezoidal functions are used to fuzzify the input data, as it is considered that there will be a range of values that might be categorized according to different ranking scales, always with several values having a full membership to each one of those scales. Triangular functions are discarded because there is not any single value, and Gaussian functions are discarded too because the attenuation they provide is not considered necessary. The same reasoning is made for the output data of both systems. Both in the case of the technical risk and in the case of the expert risk, these are subjective and appreciative values that are quantified by means of a valuation scale in the interval [10, 100]. Considering this, their membership functions are defined as trapezoidal because of the previously explained reasons, i.e., so that a range of values exists in each ranking scale, and that between them there will be always an interval—and not an individual datum—of maximum membership.

With respect to the combination rules, an analogous structure is maintained between both systems: technical and expert. An AND-type operator has been chosen for the combination and the evaluation of the membership functions corresponding to the input data, as the determination of the consequent needs of the simultaneous participation and combination of all the antecedents. The elaboration of rules will follow a non-conditioned recommendation, creating rules that are reasonable but not specific or particular to a conditioned value judgment. After this, it is proceeded to the determination of the consequent of each one of the rules obtained by using a graphical implication method which, in this case, starting from the minimum value selected for each antecedent function—after applying the AND operator—truncates at this value the consequent function. After that, the aggregation is made of all the consequent functions by means of a disjunctive approach [58] using a maximum operator that aggregates the consequents producing a final output that is the combination of the graphical envelope of all these truncated consequents. The final value for both the technical risk and the expert risk is obtained by defuzzifying the global aggregated consequent by means of the centroid method.

Even though the design of the methodology itself takes into account a re-definition of the variables and membership functions depending on the degree of application of the accumulated experience in its operation, Table 3 and Table 4 show the initial configuration of the inference systems used for the calculation of the technical risk and the expert risk within the concurrent expert systems. This initial configuration allows applying the methodology with the already described considerations, while at the same time being useful as a model to be followed in the future evolution and adaptation of the methodology.

##### Behavior of the Expert Risk Parameter

The technical risk is a non-interpretation-required parameter, and because of that the value obtained from the technical inference system may be directly used. The expert risk, on the contrary, is a fully different matter, as it comes from a qualitative and interpretative assessment [9]. Even if the presence of uncertainty in the determination of the technical risk is recognized, it is acceptable to consider that this parameter, derived from reliable information, must be considered as a plausible, effective, and decisive metric. The inference system that determines the technical risk value incorporates in itself a metric for uncertainty supervision, associated to the likelihood of error in the measurements due to the failure, or bad use, of the sensors. Because of that, the technical risk will be decisive, but always will be under the supervision of the healthcare expert. As autonomous decision-making processes are not likely to exist, especially in the healthcare field that this methodology is aimed to the judgement and criteria expressed by the medical expert not only will provide the data input for the second expert system, thus determining the ulterior expert risk, but also one of these inputs will be a qualitative valuation of the results coming from the independent and autonomous calculation of the technical risk value. Therefore, the influence of the expert risk on the global risk value, that might be assimilated to a regular medical diagnosis, is necessarily dependent on the technical risk according to the developed methodology.

Consequently, it is necessary to commit the expert’s decision, represented by the expert risk value, to be consistent with the technical risk value, taken as a reference value, avoiding in this way conditioning or falsifying the objectivity of the final decision. The expert risk value will have an influence in the global hypoxemic risk by means of what has been defined as the decision factor, that is also dependent on the technical risk, as it has been previously presented in Equation (1). 

The decision factor consists in a mathematical function of both the expert risk and the technical risk, allowing the representativeness in the expert judgment decision to be influenced not only by the expert opinions, but also by those produced by the technical risk. Thus, if both technical and expert risks are measured in a percentage scale from 10 through 100 and a middle point is established in 50, some relations are defined as a function of Equation (1) as it is mentioned in the article of the authors in Casal-Guisande et al. [9]. In summary, it is assumed that:

- When the technical risk value is less than or equal to 50, a high decision factor value will be required to obtain a high global value risk, as a result of applying Equation (1). That is why as the expert risk value raises, the decision factor value must also grow fast. This means that if the technical risk values do not indicate an alert, the expert will be responsible in exclusive of raising her valuation of the hypoxemic seriousness level by means of her assessment, which directly will result in a high expert risk value.
- If the technical risk value is greater than 50, then it will not be required the decision factor value to be as high as before to obtain a high global hypoxemic risk value, and thus high global hypoxemic risk values may be obtained with smaller expert risk values. From this, it is derived that, in situations where the technical risk value is high, the alert will prevail on the expert’s considerations, thus forcing her to justify any low evaluation results that reduce the potential alert level.

From the introduction already made, the curves have been selected that allow to implement the desired behavior among the expert risk, the technical risk and the decision factor. In this case, the use of exponential and logarithmic curves is maintained to relate the expert risk and the decision factor for technical risk values lower than or equal to 50, and higher than 50, respectively. In summary, the equations that relate to the exponential and logarithmic curves are the following: 

Exponential curves:

(2) $$f\left(R_{T},R_{E}\right)=f_{1}{\left(R_{T}\right)}^{\left(R_{E}-f_{2}\left(R_{T}\right)\right)}+10\;\forall \;R_{E,}R_{T}\;\in \left[10,\;100\right]$$

(3) $$\;f_{1}\left(R_{T}\right)=-0.0015\cdot R_{T}+1.1391$$

(4) $$f_{2}\left(R_{T}\right)=-0.0085\cdot R_{T}{}^{2}+0.5092\cdot R_{T}+57.247$$

Logarithmic curves:

(5) $$f\left(R_{T},R_{E}\right)=f_{3}\left(R_{T}\right)\cdot ln\left(R_{E}\right)+f_{4}\left(R_{T}\right)\;\forall \;R_{E,}R_{T}\;\in \left[10,\;100\right]$$

(6) $$\;f_{3}\left(R_{T}\right)=215.3\cdot R_{T}{}^{-1.005}$$

(7) $$f_{4}\left(R_{T}\right)=-0.0048\cdot R_{T}+0.7849\;$$

**Figure 4:**
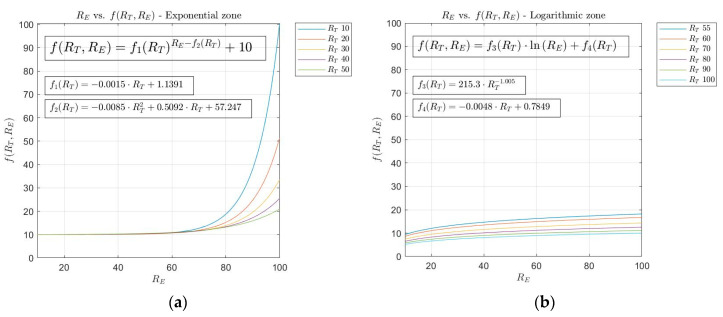
(**a**) Exponential and (**b**) logarithmic fundamental curves.

Equations (3), (4), (6) and (7) have been determined and fitted using the generalized reduced gradient method [61], on the basis of the optimization of the curves that were proposed for each value of the technical risk within the pertinent interval. Graphs (a) and (b) in Figure 4 show, respectively, the exponential and logarithmic curves.

##### Corrections

The determination of the technical risk and the expert risk values carries in itself an inherent supervision of the associated uncertainty, and therefore of the subjectivity, vagueness, and inaccuracy both of the data and of the medical assessments. However, the authors consider that in a medical application it is necessary to provide the methodology with an additional process that might be defined as ‘corrective’, which would allow a qualified expert to modify the final results obtained, i.e., a quasi-coercive measure of the appropriateness of the treatment. This process aims specifically to extend the usability of the methodology and to provide an additional supervision layer to increase the reliability of the decision finally made. Unlike in the curves model, these corrections to the determination of the risk values are specific to each medical application that the methodology is aimed at. That is so because the risk value, understood as a metric for the seriousness of a disease or a symptom, always shows a remarkable subjective component. The healthcare team in charge of the supervision and execution of the methodology must not only evaluate the results obtained, but also adapt and correct them depending on the case to be evaluated, in this case being a potential hypoxemic case. The authors in their work Casal-Guisande et al. [9] carry out a set of corrections adapted to the treatment of wounds that cannot be extrapolated to other treatments or medical diagnosis procedures.

##### Corrections on the Decision Factor Definition Curves

It might happen that the curves defined in the model are not adaptable to all the possible cases, and because of that a functionality is proposed for performing corrections on the curves to allow the expert to increase or decrease the final alert level, by modifying certain segments of the curves on the spot. Said corrections can be applied both to the exponential zone and to the logarithmic zone.

- Exponential zone: This zone applies to technical risk values less than or equal to 50. As it was explained before, while the exponential growth zone is not reached the decision factor value is almost constant. After that, once the exponential growth zone is reached, the decision factor grows fast. It might be the case that the expert wishes the growth zone to be reached before, or that a growth rate is produced that is higher than the one provided by the exponential function. In this way, the expert is allowed to perform modifications on the decision factor within a range of values of the expert risk. A new correction is proposed in this case, so that the expert has the capability to change the growth rate of the exponential function by establishing a linear transition zone in the equation that defines the decision factor. To do so, an expert risk value must be defined from which the decision factor values are intended to be modified and the desired growth rate is to be established, this rate being at all times delimited in the interval defined by $\left[\frac{f\left(R_{T},R_{E}+1\right)-f\left(R_{T},R_{E}\right)}{R_{E}+1-R_{E}},\frac{f\left(R_{T},100\right)-f\left(R_{T},R_{E}\right)}{100-R_{E}}\right]$. For example, in the case that the expert wishes to perform a correction starting for expert risk values higher than 30 with a maximum growth rate, the expression to be used is shown in Equation (8), that in essence represents a straight line.
  (8) $$f{\left(R_{T},R_{E}\right)}_{corrected}=f\left(R_{T},30\right)+\frac{f\left(R_{T},100\right)-f\left(R_{T},30\right)}{100-30}\cdot \left(R_{E}-30\right)$$
- Logarithmic zone: In the first segment of the expert risk, for values up to 30–40, decision factor values are produced that cause the global hypoxemic value to be lower than the technical risk. That is why a correction is proposed aiming to avoid a potential undervaluation on the side of the expert that might avoid the generation of warning or emergency alert levels when the expert is not fully convinced of it. When percentage variations exist between the technical risk and the global hypoxemic risk larger than a threshold value, for example a 15%, the expert will be asked about how sure she is about her assessment, and the decision factor value will be corrected according to Equation (9).
  (9) $$f{\left(R_{T},R_{E}\right)}_{corrected}=f\left(R_{E}\right)\cdot \frac{100}{\%\;Security}$$

It is relevant to say that the maximum value for the global hypoxemic risk will be 100, no matter what the decision factor value is.

#### 2.2.3. Determination of the Global Hypoxemic Risk and the Alert Level

Once the system has been defined, it is possible to perform the calculation of the decision factor, and eventually of the global hypoxemic risk, as a combination of the technical risk and the expert risk values, as it was established in Equation (1).

**Figure 5:**
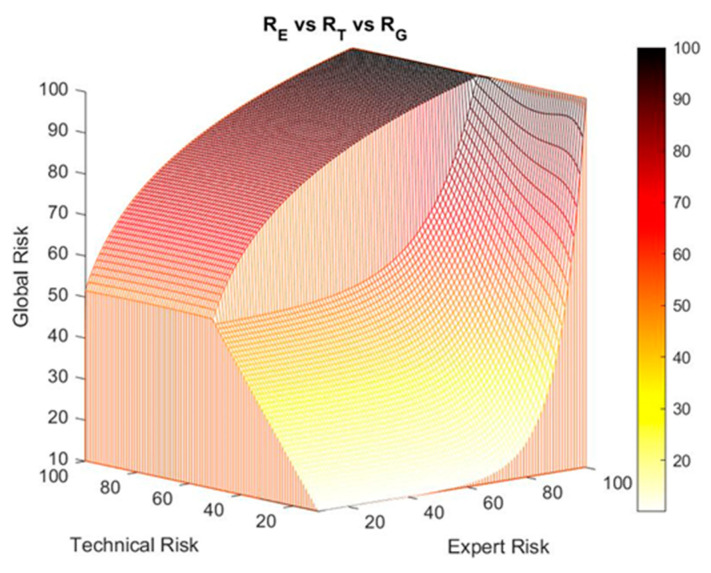
Technical risk vs. expert risk vs. global risk.

Figure 5 shows the surface of the global risk versus the expert risk and the technical risk, where the two previously described zones—the exponential and the logarithmic—are shown. 

##### Corrections on the Exponential–Logarithmic Transition Zone of the Global Risk Surface

It might be the case that the defined global risk surface does not adapt to a particular case, and because of that it is proposed to the expert the possibility of modifying said initial surface by establishing a transition zone between the exponential and the logarithmic zones. This aims to smooth the transition between both zones by connecting them, with a ramp function in this case, representing a transition that adapts to that intention and that responds to the patterns observed in reality, derived from the representability and the usefulness of the risks obtained by the system. Other transition surfaces might be proposed, provided that they represent a more appropriate and realistic behavior model. Therefore, the ramp function makes it possible to establish an alternative transition. In essence, the border between the exponential and logarithmic behavior zones is modified, avoiding sudden changes that might be translated as erroneous alert states. For this, the expert must define a range of technical risk values $\left[R_{T1},R_{T2}\right]$ inside which the correction is to be performed, with the value ‘50’ contained in that interval. Such correction interval presents maximum and minimum values for the technical risk of 75 and 25 respectively, to avoid the system losing the previously described exponential and logarithmic behaviors. The global risk value, corrected for a technical risk value $R_{Tj}\in [R_{T1},R_{T2}]$ and an expert risk value $R_{Ei}$ is shown in Equation (10).

(10) $$R_{G\;corrected}\left(R_{Tj},R_{Ei}\right)=R_{G}\left(R_{T1},R_{Ei}\right)\;+\frac{\left[R_{G}\left(R_{T2},R_{Ei}\right)-R_{G}\left(R_{T1},R_{Ei}\right)\right]}{R_{T2}-R_{T1}}\cdot \left(R_{Tj}-R_{T1}\right)$$

**Figure 6:**
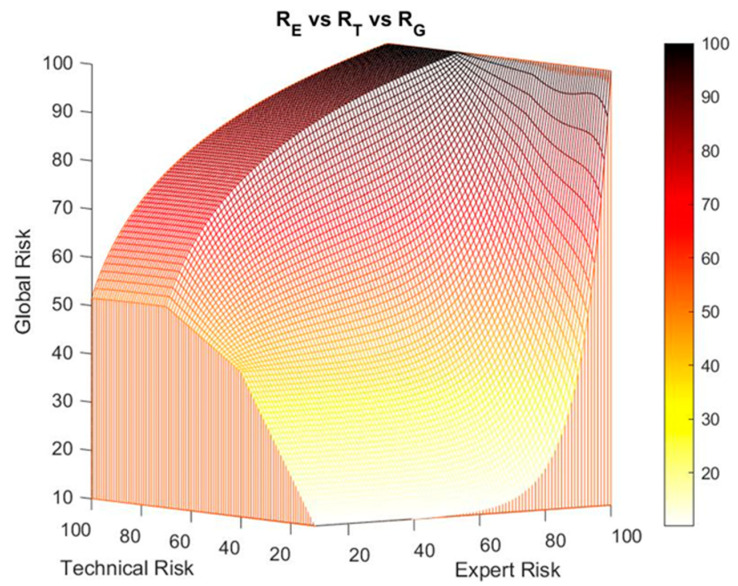
Example of a ramp correction: technical risk vs. expert risk vs. global risk.

Figure 6 shows the results after performing a second-level correction within the technical risk range between the values 40 and 70. This correction on the global risk surface is compatible with the first-level correction on the decision factor curves that was formerly introduced, keeping the highest global risk value obtained. For example, if a first global risk value of 90 is obtained from the first-level correction and a second value of 80, the first value will prevail over the second one. 

#### 2.2.4. Alert Evaluation

The last stage in the methodology consists in the assessment of the system states by means of the interpretation and evaluation of the results obtained, represented by the global risk value. On the basis of a set of pre-defined values, the possible states of the system are as follows:

- Wait: when the global risk value is contained in (0–60).
- Warning: when the global risk value is contained in (60–80).
- Emergency: when the global risk value is contained in (80–100).

From those assessment results the necessary actuations will be established by the emergency services. The pre-defined limits can be modified at any given time according to the experience gained and the accumulated data.

## 3. Simulation and Results

### 3.1. Simulation

Once the methodology was defined, an implementation of it was carried out by using the MATLAB© R2020a [60] software environment.

On the basis of such implementation, different tests were carried out to check the effectiveness and validity of the methodology, and also to guarantee the effective generation of alerts and the calculation of all the terms involved in the determination of the global risk value. The data input to the system for the different tests were provided by a healthcare team, and are shown in Table 5. Said data is associated with 30 patients and aims to be representative of different initial circumstances, such as: age, prior conditions, surgical procedures, etc. At it has been already explained at the beginning of Section 2.1.2, the use of the methodology proposed is cross-sectional, meaning that it will assess the hypoxemic risk derived from multiple causes. Because of that, it will not be focused on a particular condition or on an alert situation such as the COVID-19 pandemic, but these factors will represent an additional set of criteria to be considered. All the appropriate privacy protocols have been followed across the whole process, and the data may be distributed without harm to any person, collective, or institution, since its use has been granted for training or academic research purposes.

Initially, and for the sake of the demonstration of the operation of the methodology, patient 1 of Table 5 is to be analyzed in detail, with the initial data coming from the sensor readings being as follows:

- Oxygen blood level: 92%.
- Heart rate: 80 beat/min.
- Temperature: 37 °C.

**Figure 7:**
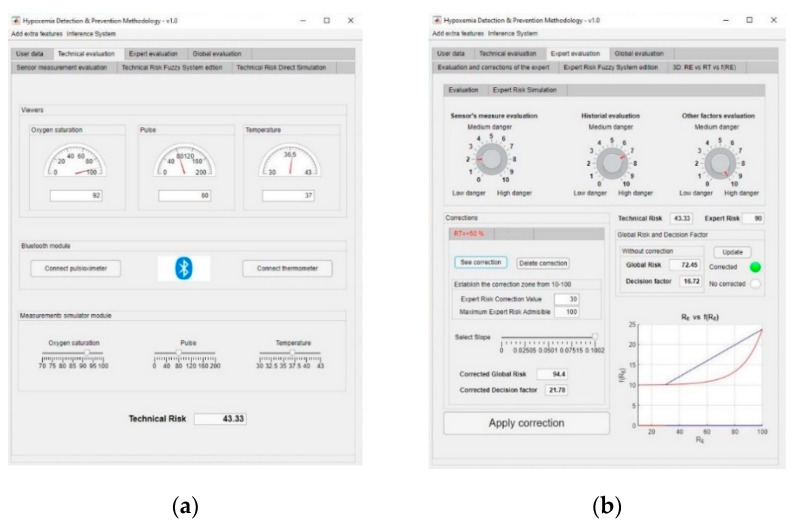
(**a**) Technical evaluation dashboard; (**b**) expert evaluation dashboard.

This information is fed into the application as shown in Figure 7a, and thus a technical risk value of 43.33 is obtained.

Subsequently, the expert performs an assessment of the collected measurements, the patient’s history and all the other potential risk factors that were previously introduced by the patient into the application. In this case, the expert makes the following considerations:

- Assessment of measurements: medium-low risk level (3/10).
- Assessment of the patient’s medical history: In this case the risk level is medium-high, as the patient is affected by sleep apnea and chronical obstructive pulmonary disease (COPD) (7/10).
- Assessment of any other potential risk factors: The risk is high, as the patient has an at-risk job (10/10).

Once the evaluation has been carried out, its results are introduced into the application, as shown in Figure 7b, and thus an expert risk value of 90 is obtained.

From the duple (technical risk, expert risk) a specific value for the decision factor is obtained, in this case being of 16.72, which produces a global risk value of 72.45.

It might be the case that the expert wishes to perform a correction on the curves that define the decision factor value, i.e., a first-level correction. In this case, the expert establishes a value of expert risk and a slope to perform the correction. Thus, a corrected decision factor value of 21.78 is obtained.

**Figure 8:**
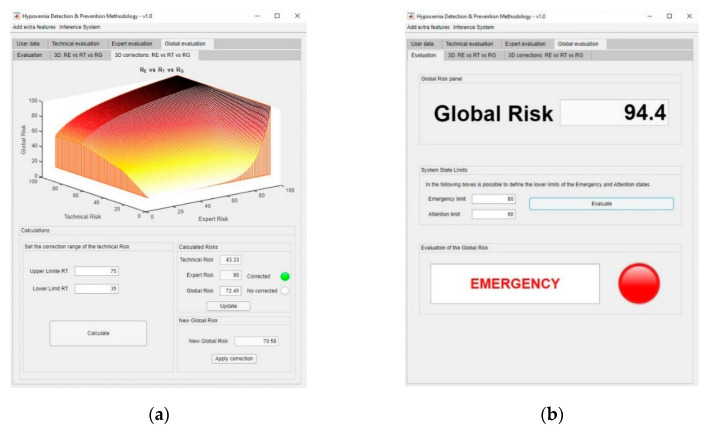
(**a**) Sigmoidal correction dashboard; (**b**) final classification and alert level.

In the same way, it is possible to apply a ramp correction, independent from the first-level one, as a second-level correction on the global risk surface, if the expert considers it to be convenient. Figure 8a shows the dashboard used for the definition of the second-level corrections on the global risk surface. Initially, the global risk value was 72.45, indicating a warning status. After the first- and second-level corrections, global risk values of 94.4 and 70.58 were respectively obtained, implying an emergency state, as the higher risk obtained after the correction prevails. In this case, it will be recommended to immediately send an emergency service team to the patient’s, as shown in Figure 8b.

### 3.2. Results

Before performing a more detailed analysis of the results obtained by comparing them to those resulting from a conventional diagnosis process, it is necessary to carry out a more precise assessment of the use and meaning of the presented methodology in the field of the early detection of respiratory insufficiency cases associated to hypoxemic conditions. The risk values obtained in the case study are consistent with the collected data and with those derived from the traditional diagnosis approaches. However, the fluctuation in the global risk value that depends on the different correction levels integrated into the methodology being applied might appear incoherent at a first sight. However, this is only an appreciation derived from the direct comparison. When the true nature of a diagnosis process is taken into consideration, it is observed that even if it might be helped by a decision-support methodology, it will always include and contemplate a wide variety of direct and indirect criteria within its development. An effective healthcare evaluation must minimize the risk for the patient’s health by means of reducing errors and uncertainty, associated to both data collection and to its interpretation. It is precisely in this sense where the supporting methodologies such as the one presented in this work might be very helpful, as they reduce that uncertainty in the data chain relative to data collection, its processing, and even the decision inference made based on it. However, even if these methodologies are effective, they cannot—and must not—elude to be monitored by the expert healthcare team, which possesses an overall diagnosis view supported by the experts’ experience and training. Consequently, the fluctuation in the risk values derived from the application of rules is not a handicap of the methodology, but it is rather one of its most remarkable features. In a highly complex situation such as the one surrounding a diagnosis process, expert systems may help to infer decisions, but they will always depend on their knowledge base and on the design of their inference engines. That is why in the case of the presented methodology the subsequent corrections aim to, on the one hand, correct and monitor the recommendations provided by the methodology, and on the other hand begin incorporating such corrections in a way that they get integrated into the expert systems’ knowledge base, so that in each subsequent application they can obtain more reliable decisions. They are, therefore, a reflection of—and at the same time an answer to—the high degree of complexity in the diagnosis process.

Changing the subject now to the comparative analysis of the results, in Table 6 a summary is shown of the different simulations for the patients from Table 5. It is important to mention that the global risk values shown in this table were calculated without performing any correction on them. The last two columns present the state recommended by the system and the actual status of the patients. It is also relevant to mention that the expert healthcare team who evaluated these cases did not know the actual status of any patient, in order to avoid any previous conditioning to their assessment. In Table 6, it is considered that the emergency state also includes the warning state, so that such emergency state is considered for global risk values greater than or equal to 60, as was established in the own definition of the methodology.

In Table 7, a summary of the results of the simulation carried out is presented.

It is proceeded then to the calculation of the method’s accuracy metric, this understood as the ratio of the well-classified cases to the total cases (see Equation (11)) [62]. In this case an accuracy value of 76.67% is obtained, that is, 7.7 out of 10 patients were correctly classified.

(11) $$Accuracy=\frac{t_{p\;}+t_{n}}{total}=\;\frac{16+7}{30}=76.67\%$$

In order to guarantee the validity and usefulness of the data, these were characterized by their specificity and sensitivity values. Equations (12) and (13) respectively show their mathematical expressions. Basically, the specificity metric evaluates the usefulness of the test in order to identify negative cases, while the sensitivity metric indicates how good the test is at determining the positive cases [62]. In this case, a specificity value of 53.85% and a sensitivity value of 94.12% are observed.

(12) $$Specificity=\frac{t_{n}}{t_{n}+f_{p}}=\;\frac{7}{7+6}=53.85\%$$

(13) $$Sensitivity=\frac{t_{p}}{t_{p}+f_{n}}=\;\frac{16}{16+1}=94.12\%$$

From these values it is possible to calculate the fraction of false positive and false negative cases (see Equations (14) and (15), respectively). It is observed that the fraction of false negative cases was 5.88%, while the fraction of false positive cases was 46.15%.

(14) P(false negative)=1−sensitivity=5.88%

(15) P(false positive)=46.15%

With the purpose of evaluating the overall effectiveness of the method, the calculation of the Youden index, proposed in 1950 [63], was carried out (see Equation (16)). In this case a value of 0.4797 was obtained. According to this index, the method gets better as the index value gets closer to one.

(16) Youden index=sensitivity+specificity−1=0.9412+0.5385−1=0.4797

It is necessary to highlight that the state recommended by the system is mostly determined by the expert’s opinion and by the established global risk limits that define the different states. That is why this index may be useful to evaluate the method and the expert itself as a whole.

**Figure 9:**
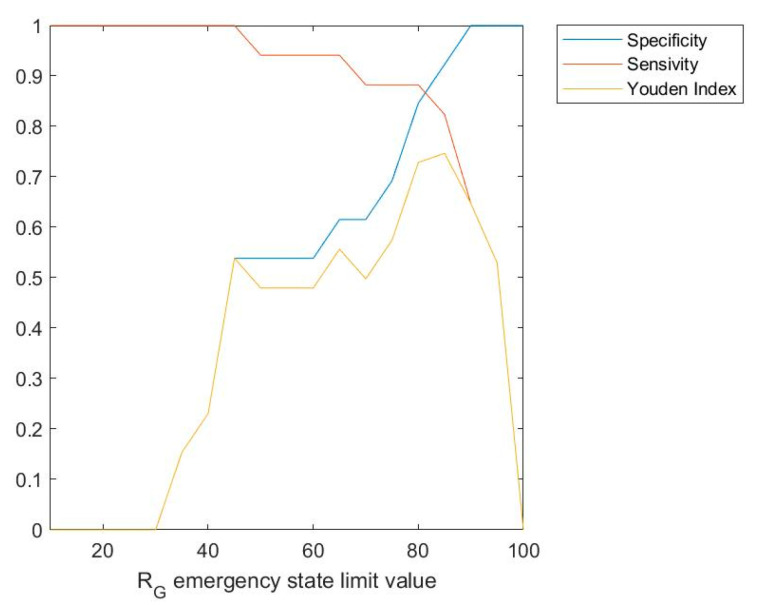
Sensitivity, specificity, and Youden index vs. emergency state limit value.

Figure 9 shows a graph in which the sensitivity, specificity, and Youden index values, versus the limit value for the emergency state are represented. As it can be observed in the figure, Youden index is maximized for a global risk limit value for the emergency state of 85, at which a value of 0.746 is obtained. That is why the calculation of these values may help to determine which should be the global risk limit value for the emergency state for each particular expert.

Additionally, and with the purpose of evaluating the reliability and reproducibility of the recommendations proposed by the methodology, the kappa coefficient proposed by Jacob Cohen in the 1960s [64] was also calculated. Such coefficient allows to independently calculate the stability and concordance of a classification from a sampling made by two ‘judges’, which in this case are the methodology and the healthcare team. Equation (17) shows the definition of this coefficient [64] and the result of its calculation for the case study, with $p_{o}$ being the ratio of sample units which the judges agree upon, and $p_{c}$ the ratio of units for which the concordance is due to chance or randomness. The $1-p_{c}$ term reflects the disagreement between the judges. The $p_{o}-p_{c}$ term represents those cases in which the concordance is caused by factors not associated with chance or randomness. In relation with the interpretation of the kappa coefficient, total agreement is equivalent to a kappa value of 1, while an agreement due purely to chance or randomness is associated to a kappa value of 0 [65]. In this case, for a kappa value of 0.502, it may be concluded that a moderate agreement level has been obtained [65].

(17) $$\kappa =\frac{p_{o}-p_{c}}{1-p_{c}}≅0.502$$

**Figure 10:**
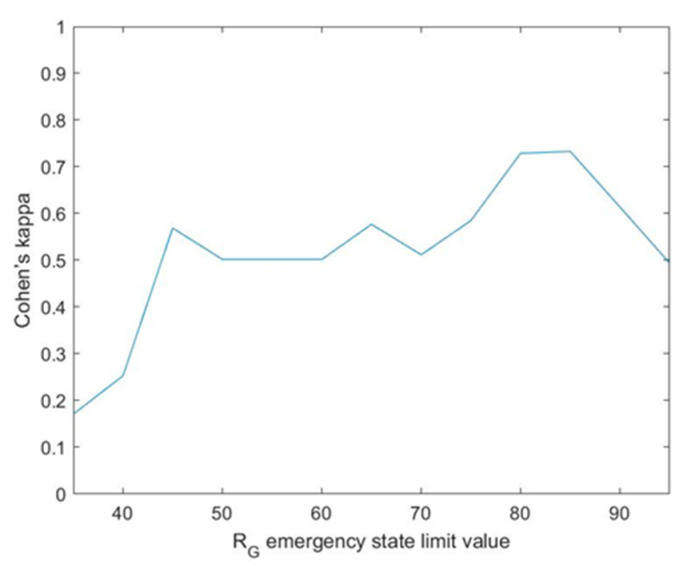
Cohen’s kappa vs. emergency state limit value.

The graph shown in Figure 10 represents Cohen’s kappa coefficient versus the emergency limit. As it already happened in the previous graph, the kappa coefficient value reaches a maximum for a Global Risk value of the emergency state of 85, reaching a value of 0.733. This may be interpreted as a substantial agreement [65]. In line with the interpretation comments of Figure 9, the availability of Figure 10 helps to strengthen what is the most appropriate limit value for the determination of the emergency state.

#### Interpretation of the Results

Several metrics were selected in order to evaluate the results obtained. Accuracy, specificity, and sensitivity are referred to the evaluation of a binary classifier, in this case the emergency or non-emergency state. In the case of the accuracy, the best case scenario would be to obtain a 100% value, meaning that all the patients, both those associated to the emergency and to the non-emergency states, were correctly classified. In this case, an accuracy value of 76.67% was obtained. In relation to the specificity, the metric that in this case evaluates the capability for detecting the non-emergency state, a value of 53.85% was obtained, with the best case being a value as close as possible to 100%. The objective of the sensitivity in this case is to assess the capability for detecting the emergency state, with a value of 94.12% being produced that is very high and close to 100%. The Youden index can conjointly evaluate specificity and sensitivity, implying better results as the value obtained gets close to 1. In this case a Youden index value of 0.4797 was obtained, mainly because of the moderate value of the specificity.

Cohen’s kappa index was also calculated, aiming to show a measurement of the correlation that exists between the recommendations of the methodology and those other proposed by the healthcare team. The closer this index value gets to 1, the higher the concordance between those two recommendation sets will be. A value of 0.502 was obtained for this kappa index, thus pointing out to a moderate agreement level.

If Figure 9 and Figure 10 are taken into account, there is no doubt that the limit to the global risk that is associated to the emergency state must be reviewed in this case. Better results have been observed for a limit of 85, for which the Youden index value grows from 0.4797 to 0.746 and Cohen’s kappa does as well from 0.502 to 0.733, which brings together an improvement on the accuracy, the sensitivity and the specificity metrics, and at the same time of the agreement level between the methodology and the healthcare team.

It is unquestionable that the choice of the emergency limit value has a great impact on the decisions of the methodology, and thus it must be reviewed and calibrated by a specific expert before its final deployment.

From a safety viewpoint, it is preferable that the methodology shows moderate specificity values, provided that it ensures obtaining high sensitivity values, as close to 1 as possible. In the quest for balancing the sensitivity and specificity values, a global risk value of 85 is postulated as being a very good option in this case for setting the emergency limit at.

Even if the experimental nature of the presented methodology must be highlighted, it is worthy to point out that the use of expert systems as facilitators for the decisions associated to medical diagnosis results to be a tangible and steadily-growing reality. The results obtained, both from the point of view of diagnosis and from the practical one, invite to optimism. The recommendations derived from the application of the methodology, even if they disagree with the actual ones because of the previously explained reasons, recognize all of the most damaging and dangerous cases for the patient’s health. This does not fully imply its reliability, but only indicates a trend that energizes the evolution of the methodology.

## 4. Discussion

The methodology developed in this article allows to minimize the diagnosis time for potential hypoxemic-related medical cases, and it is expected to allow to reduce the potential impact that these cases cause on the health of the affected patients. The reduction in the time needed and in the number of errors produced in the diagnosis process is one of the key objectives of the decision-support systems aimed to be used in medical environments. This is even further reinforced in pandemic situations, such as the current one associated to COVID-19, where the agile and early detection of respiratory insufficiency cases is essential. In order to achieve this goal, a process was established, structured in several stages, that starts from heterogeneous information coming from both sensor data collection and from the manual input by the patient. Such information is then fed into a decision-support system composed of two Mamdani-type fuzzy logic-based expert systems working concurrently. These allow obtaining the technical risk and expert risk values, according to the provided definitions, values that are needed to calculate the hypoxemic global risk. From this last value it is possible to generate the alerts relative to the need for the intervention of the health services.

As it has been introduced before, it is every time more common to have available reliable network and data structures, which facilitate the growth and development of new monitoring and diagnosis methods. By combining the use of measurement devices with fuzzy-logic inference systems, the software artifact that implements the methodology has the capability to process the collected information and to recommend decisions based on such information, thus speeding up even more the diagnosis process. Even if the innovation proposed by the authors in the 2020 article [9] was already justified and compared to the state of the art in its field of study, i.e., the decision-support systems in the medical sphere, the subsequent adaptations of concurrent expert systems applied to diagnosis procedures are in themselves a novelty to be highlighted. It is commonly known that expert systems are used in combination with decision-support systems in several different fields of governance and engineering. Their capabilities for the diversification and formalization of information are a determinant factor in their use to support decision-making processes. In this way, by means of the Mamdani’s approach and the incorporation to the abilities of the healthcare team of the correction and modelling of the behaviour of the system, a decision-support methodology was developed that shows large predictive—and therefore preventive—capabilities aimed to respiratory insufficiency clinical cases. 

The experimental results obtained have been satisfactory, inviting to think that the methodological development contemplates all those factors that are necessary for a robust and reliable early detection of potential hypoxemic cases. The expert systems help to reduce the uncertainty derived from the diagnosis process from the random, epistemic and interaction-related perspectives, as well as the one due to the vagueness in language [59], by supervising the chain of data collection, elaboration, and processing. Moreover, the incorporation of the corrections extends the use and applicability of the methodology, as it allows the healthcare team to implement modifications on the suggestions inferred by the expert systems, thus enriching its knowledge base for future applications as well. The resulting risk values were within a range that was similar to another range, this one coming from on-site tests when taking into account exclusively the initial data, thus reinforcing the idea of the effectiveness of using concurrent expert systems. This parallelism that can be observed in the results from traditional diagnosis procedures, and the presented methodology not only contributes to reinforce the use of expert systems, but also suggests a more intensive practical application of the methodology. While some disparity in the recommendations has been observed when comparing the results of the methodology with those from a conventional diagnosis process, it has been also established that the recommendations made by the methodology are always on the safe side, and no patient in an actual state of emergency has been wrongly classified. As it has been demonstrated, a readjustment in the global risk ranges assigned to each recommendation substantially improves the statistical parameters of correspondence between those evaluations. Even if it must always be under supervision because of the critical effects of potential diagnosis errors, the comprehensive data treatment together with the capability to infer relationships among them and to predict tangible results, are determinant and differential aspects in the methodology. It is not to be disputed that its usage will not possibly be error-free; nevertheless, the expected benefits derived from the tests that were carried out, and also the need to have available fast and reliable diagnosis mechanisms, make its realisation necessary. 

The combined use of expert systems embedded into a diagnosis methodology is, at the moment, a field in development with different proposals [9,34,35,36], and which currently is undergoing a steady expansion process. Generally, the acceptation of clinical decision support methodologies is a topic that is currently under extensive research [33]. These methodologies show a great potential of use, from both the care and the economic points of view, but they display evident deficiencies regarding their applicability and reliability. Many authors, such as Miller and Geissbuller [66], claim that their use is limited to be helping tools and that they will not be able to replace the full diagnosis processes. Others, such as Pawloski et al. [67], show results that allow claiming that the clinical decision support systems make possible to improve the results of the diagnosis process. There is an evident dichotomy in the current debate about the usefulness of the decision support tools in the clinical field that the design and development of the presented methodology is not far-removed from. As it is not possible to guarantee that the support methodologies might provide unquestionable benefits, being far from becoming a possible diagnosis alternative, they must be designed taking that into account. The proposed methodology incorporates all those considerations and allows a complete and full revision of its decision-making process, from the most essential levels to those included in the management interface itself. It is about highlighting its application as a decision-support tool, its capability to process data and to reduce uncertainty, and its inference of an objective risk level, beyond a diagnosis capability that must be re-interpreted and monitored by the healthcare team.

On the other hand, it is also convenient to evaluate the impact of the proposed methodology on the early detection of hypoxemic cases. As it was already mentioned in Section 1.4, at the moment the most remarkable research lines in this field might be divided into those related to the use of blood oxygen measurement instruments and devices, and those other addressing the definition of clinical decision support systems. With regard to the advances in the use of oxygen measurement devices or pulse-oximeters, several studies and approaches [12,40,41,42,43,44] highlight the different criteria for their placement and the interpretation of the results obtained. As for the second research line mentioned, all the reviewed works [45,46,47,48,49,50,51] have been already described in Section 1.4.2. Generally, all of them propose systems that, starting with the pulse-oximeter readings, implement predictive solutions based on different technical approaches. After performing a critical comparison of the presented methodology to those already analyzed and included in the literature, it is possible to conclude the following:

- The proposed methodology allows to integrate all those aspects related with improvements in the reliability, placement and interpretation of the pulse-oximeter readings. In this case, the integration of this device is carried out by means of an inference system that fuzzifies its measurements. This makes possible to improve its qualitative interpretation. In the same way, suggestions related to its placement or to the threshold values may be directly implemented in the membership functions of the technical and expert risk antecedents, by means of the assessment of the information collected in all the related studies. They can even be reflected on the definition of the global risk ranges associated to the definition of the recommendations.
- All the works analyzed related to decision support systems aimed to the detection of hypoxemic cases are based on the integration of data and the determination of values for warning and alert thresholds. According to this approach, the determination of the global risk in the presented methodology is similar to those with the following considerations:
  ○ The global risk is a function calculated from the inference of the technical risk and the expert risk, which allows to integrate quantitative and qualitative variables into the calculation process, thus incorporating the assessment of the healthcare team.
  ○ The global risk ranges associated to the recommendations can always be modified by the healthcare team, being liable to alterations or modifications adapted to the collected dataset.
  ○ The processing of the initial data of both inference systems is performed by means of the definition of membership functions, reducing the uncertainty in the process of quantification of its qualitative expression.
  ○ The use of expert systems, a key feature of this methodology, makes possible as already explained the diversification and formalization of the information related to the detection of hypoxemic conditions, thus allowing the methodology to be used by different experts possessing different training and experience.
  ○ The incorporation of corrections, as indicated in the results section, significantly extends the usefulness of the methodology. It has already been argued in this section that the benefits of this type of support tools to the decision process is currently under question. The presence of corrections reinforces its character to support and to help the diagnosis process, but always leaving the final decision to the healthcare team, which always may re-interpret the results provided by the expert systems.
  ○ The interface, same as happens in the works by Karlen et al. [45], Keerthika and Ganesan [46], Isik and Güler [47], and Merone et al. [48], makes possible to implement a system for the application of the methodology. Even if it is has not being incorporated yet into mobile devices, such a realization would be simple. Additionally, the software artifact developed was conceived for its incorporation into clinical information systems, therefore being useful at times in which, either because of the large number of patients, or because of the widespread dissemination of a disease, said systems work at saturation conditions. A clear example of this is the health system collapse caused in many areas by the COVID-19 pandemic that has circulated worldwide in 2020.
  ○ Both the concurrence in the inference of the expert systems and the global risk calculation and the corrections, provide an effective supervision of the uncertainty. It is convenient to highlight that, further away from the risk that is evaluated here, a more essential risk exists associated to this uncertainty that measures what is unknown or what cannot be assessed. It is clear that, for all expert systems, to evaluate what is uncertain is a key point that, in this case, gets even bigger because of the particular importance of the specific topic dealt with. The methodology does not only reduce the uncertainty from the collection and interpretation of the input data, but also makes possible to handle what is uncertain by means of the correction, thus building trust in the processes.
  ○ Lastly, it must be highlighted that the presented methodology is modular, meaning that it will always be possible to incorporate it into other inference engines or prediction algorithms. This integrating capability, derived from its own design, allows other developments in the field of study to be integrated into it, if that is the case.

The methodology has been partially translated into an artifact presented as a software application that allows to establish the behavior of the two inference systems and to perform the calculations that were previously defined in this work. At the moment, the computer program that encompasses the whole methodology and the bi-directional communication protocols between the patient and the expert, as well as the ways for transmitting and interpreting the information, has not been elaborated yet. Also, in the case the methodology is implemented into a mobile application, it will be necessary to guarantee a continuous connectivity of the device so that the alert generation system may be effective.

## 5. Conclusions

The methodology proposed in this work is supported by the use of concurrent expert systems defined by means of fuzzy-logic inference systems that are capable of producing a final global risk value associated to the risk a patient has to develop a hypoxemic case. Moreover, management capabilities are set up for the uncertainty associated to the input data, both quantitative and qualitative, providing a correction system to implement the indeterminacy in the diagnosis. With all that, it is achieved to improve the medical monitoring process for the prevention of the previously mentioned cases, while reducing at the same time the time needed to perform an on-site traditional medical assessment, and minimizing the impact and the potential effects derived from a late detection in patients at risk.

The proposed methodology has been applied to a case study by means of its implementation into a software artifact. The results obtained support a very high applicability of the methodology, especially when an early and reliable diagnosis is required based mainly on subjective information, even if they cannot be considered as conclusive yet. The design of the methodology, combined with the use of expert systems, can extend its applicability when compared to the current clinical decision support systems, because it not only makes an appropriate processing of information possible, but it also incorporates tools that increase the confidence and reliability of its outcomes. All that is shown in the comparison made between the results obtained after the application of the methodology and those other collected when it is not used. Thus, even if full consistency has not been achieved, the fact that all patients in an emergency state were correctly characterized, together with the estimation of a higher correspondence of the recommendations in the presence of variations in their determination range, can estimate at least that the methodology has a clear potential to help and complement the clinical diagnosis process. In any case, a more extensive application to real environments will be necessary in order to obtain precise metrics for the actual validity of its use.

The improvement in the reliability of the recommendations must be driven by means of the improvement of both the data collection processes and the inference processes associated to the final decision. As its use as a decision-support tool is precisely the main characteristic of the presented methodology, its future performance must mandatorily involve a more extended definition of its knowledge base, which will result in more reliable results that at the same time require less interpretation. The future incorporation of hierarchization algorithms for the risk level, and the pre-treatment of data by means of non-supervised learning approaches, are future research lines that will be developed as a consequence of this work.

Finally, it is important to highlight the usefulness and the versatility of the presented methodology, as it can also be applied to the treatment of other diseases in which it is necessary to establish a global risk index as a result of the combination of assessments of different nature—in this case technical and expert—as it could be the monitoring of diabetes or heart arrhythmia, among many others.

## Figures and Tables

**Table 1:** Verification of the guidelines by Hevner et al. [13,52].

Rule 1: Design an artifact (presented methodology)
The artifact, i.e., the methodology detailed in Section 2.2, is a tool aimed to help in the process for assessing the health status of patients prone to developing respiratory diseases.
Rule 2: Relevance of the problem
The assessment of the health status of patients liable to develop potential medical hypoxemia cases is nowadays a topic of vital importance, of undoubtable relevance because respiratory diseases are globally the fifth most relevant cause of death [1,2], and more especially during 2020 due to the pandemic situation caused by COVID-19.
Rule 3: Design evaluation
The application of a new methodology is demonstrated in the practical case shown in Section 3.1.
Rule 4: Contributions to the field of research
The contributions to the field of new expert systems applied in the context of decision-making support in medicine are presented in Section 4 and Section 5 of this article.
Rule 5: Rigor in the research
The conceptual development of the presented methodology has been defined in Section 2.1, as was its classification within the field of research. In the same way, the mathematical groundings of this work are supported on the use of fuzzy-logic inference systems, and the definition of the risk functions and their combinations has adhered at all times to the appropriate mathematical rigor.
Rule 6: Design as a search process
In Section 1, the methodology was framed within the state of the art of similar applications, observing its differential aspects. These differences are discussed in Section 4.
Rule 7: Communication of the research
In Section 4 and Section 5, the main contributions of the new method are presented, as well as the future lines of work.

**Table 2:** Questions to be answered periodically by the patient.

Name:	Identifier:		
Date of Birth:	Race:	Sex(M/F):	
Questions to Be Answered:		Yes	No
Have you been abroad in the last month?			
If yes, please, name the region where have you been.			
If yes, for how long, in months, have you been abroad?			
Have you had any risk contact with people affected by—or that might be affected of—COVID-19?			
Have you suffered—or are you suffering now—of fever, dry cough, or fatigue?			
Have you had—or do you have now—breathing difficulties or a shortness of breath feeling?			
Have you been—or are you now—affected by loss of smell or taste, headache, or any other discomfort or pain?			
Do you have an occupation that involves risk to the respiratory system—for example, participation in mining operations?			
If yes, please, explain which occupation it is			

**Table 3:** Initial configuration of the technical risk inference system

Technical Risk Fuzzy Inference System.			
Input data	Range	Output data	Range
Oxygen saturation	70–100%	Technical Risk	0–100 ^1^
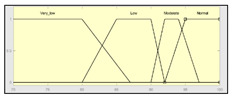		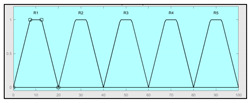 ^1^ Actual range is 10–100, for more details see Section 2.2.1.	
Temperature	33–41 °C	Initial configuration	
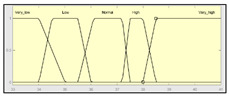		Fuzzy structure: Mamdani–type. Membership function type: trapezoidal. Defuzzification method: centroid [58].	Implication method: MIN. Aggregation method: MAX. Number of fuzzy rules: 45.
Heart rate	15–180 b.p.m	Subset of the 45 fuzzy rules 1. IF (O2_Saturation is Very_Low) AND (Pulse is Normal) THEN (Technical_Risk is R5) 2. IF (O2_Saturation is not Very_Low) AND (Temperature is Normal) AND (Heart_Rate is Normal) THEN (Technical_Risk is R1) 3. IF (O2_Saturation is not Very_Low) AND (Temperature is Normal) AND (Heart_Rate is High) THEN (Technical_Risk is R1)	
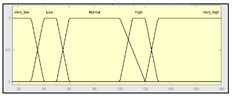			
Example of combination of fuzzy rules 2 and 3			
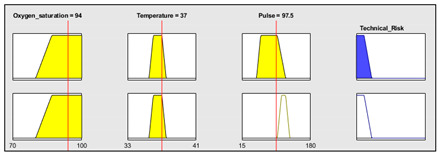			

**Table 4:** Initial configuration of the expert risk inference system.

Expert Risk Fuzzy Inference System			
Input data	Range	Output data	Range
Sensors’ measurement assessment	0–10	Expert Risk	0–100 ^2^
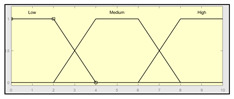		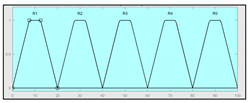 ^2^ Actual range is 10–100, for more details see Sub-Section 2.2.1.	
History assessment	0–10	Initial configuration	
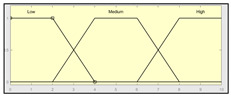		Fuzzy structure: Mamdani–type. Membership function type: trapezoidal. Defuzzification method: centroid [58].	Implication method: MIN. Aggregation method: MAX. Number of fuzzy rules: 29.
Assessment of other factors	0–10	Subset of the 29 fuzzy rules 1. IF (Sensors_Measurement_Assessment is Medium) AND (History_Assessment is Low) AND (Assessment_Other_Factors is Low) THEN (Expert_Risk is R1) 2. IF (Sensors_Measurement_Assessment is Low) AND (History_Assesment is High) AND (Assessment_Other_Factors is High) THEN (Expert_Risk is R5)	
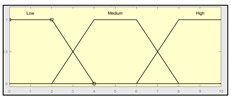			
Example of combination of fuzzy rules 1 and 2			
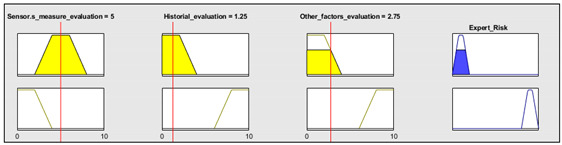			

**Table 5:** Patients’ data.

Patient	O_2_ Conc.	Heart Rate (beat/min)	Temp. (°C)	History	Other Factors
1	92	80	37	55 y.o., sleep apnea and COPD	Mining job
2	87	50	38.5	60 y.o., smoker and sedentary	-
3	80	60	37.1	47 y.o., lung cancer	-
4	93	140	38	18 y.o., obesity and asthma	-
5	83	80	39	78 y.o., ex-smoker	-
6	91	96	37.8	24 y.o.	-
7	95	56	36.5	15 y.o., asthma	-
8	90	72	36	35 y.o., smoker	Stone work job
9	89	55	35.9	93 y.o., ex-smoker	-
10	75	50	38.5	70 y.o., lung oedema	-
11	96	64	36.5	25 y.o.	-
12	89	74	36.6	26 y.o., smoker	Risky contacts
13	92	56	37	45 y.o., sporty	-
14	87	83	37.1	44 y.o., post-surgery	-
15	80	63	35.8	92 y.o.	-
16	65	50	35.8	87 y.o., palliative care	-
17	86	92	37.2	17 y.o., obesity	-
18	95	72	36.6	49 y.o.	-
19	74	63	35.9	50 y.o., alcoholic and smoker	-
20	93	89	37	23 y.o.	-
21	89	66	36.7	67 y.o., ex-smoker and sedentary	-
22	82	70	37.2	52 y.o., post-surgery	-
23	92	68	36.3	84 y.o., sporty	-
24	70	51	35.8	77 y.o., lung cancer	-
25	89	66	36.3	36 y.o., asthma	-
26	89	66	36.3	36 y.o., asthma	-
27	87	94	37.2	59 y.o., COPD	-
28	90	56	36.9	43 y.o.	-
29	96	71	36.7	38 y.o., smoker	-
30	82	84	36.4	66 y.o., sleep apnea	-

**Table 6:** Comparative of the recommended and actual state of the patients.

Patient	R_T_	R_E_	R_G_	Recommended State	Actual State
1	43.33	90.00	72.45	Emergency	Non-emergency
2	80.00	74.40	94.00	Emergency	Emergency
3	90.00	90.00	97.92	Emergency	Emergency
4	61.84	53.62	87.01	Emergency	Non-emergency
5	81.47	90.00	98.01	Emergency	Emergency
6	45.11	10.00	45.25	Non-emergency	Emergency
7	38.59	10.00	38.67	Non-emergency	Non-emergency
8	57.21	40.16	80.84	Emergency	Emergency
9	55.93	69.08	92.28	Emergency	Emergency
10	90.00	90.00	97.92	Emergency	Emergency
11	34.10	34.13	34.13	Non-emergency	Non-emergency
12	56.67	90.00	97.85	Emergency	Emergency
13	43.72	40.46	44.59	Non-emergency	Non-emergency
14	56.67	90.00	97.85	Emergency	Emergency
15	90.00	90.00	97.90	Emergency	Emergency
16	90.00	90.00	97.90	Emergency	Emergency
17	57.62	40.00	80.76	Emergency	Non-emergency
18	40.68	10.00	40.75	Non-emergency	Non-emergency
19	90.00	90.00	97.90	Emergency	Emergency
20	43.33	10.00	43.43	Non-emergency	Non-emergency
21	56.67	31.33	75.59	Emergency	Non-emergency
22	65.67	56.06	87.98	Emergency	Emergency
23	43.33	29.66	43.72	Non-emergency	Non-emergency
24	90.00	90.00	97.70	Emergency	Emergency
25	56.67	22.51	68.61	Emergency	Emergency
26	56.59	40.00	87.74	Emergency	Emergency
27	55.92	16.33	61.82	Emergency	Non-emergency
28	33.47	20.00	33.54	Non-emergency	Non-emergency
29	64.63	56.78	88.24	Emergency	Emergency
30	52.83	35.94	78.41	Emergency	Non-emergency

**Table 7:** Simulation results.

Binary classification groups	Classification state	Number of Cases
Correctly classified patients	True positive (t_p_)	16
	True negative (t_n_)	7
Wrongly classified patients	False positive (f_p_)	6
	False negative (f_n_)	1
